# ASSOCIATIONS BETWEEN HURRICANE-RELATED STRESSORS AND PERCEIVED STRESS IN A SAMPLE OF PUERTO RICANS

**DOI:** 10.1093/geroni/igad104.0849

**Published:** 2023-12-21

**Authors:** Erin Ballard, Victoria Guinn, Sheila Thompson, Ross Andel, Lisa Brown, Michael Crowe

**Affiliations:** University of Alabama at Birmingham, Birmingham, Alabama, United States; Palo Alto University, Palo Alto, California, United States; Palo Alto University, Palo Alto, California, United States; University of South Florida, Tampa, Florida, United States; Palo Alto University, Palo Alto, California, United States; University of Alabama-Birmingham, Birmingham, Alabama, United States

## Abstract

This project examines the relationship between hurricane-related stressors and perceived stress using data from the latest (third) wave of the Puerto Rican Elder: Health Conditions (PREHCO) study. A total of 617 PREHCO survivors, age 78 and older, were asked questions about their experience with stressors related to hurricane Maria. We assessed self-reported fright felt immediately after the hurricane as a primary stressor. A list of secondary stressors was examined using a principal components analysis (PCA) that yielded three components related to concern over home damage, family/friends, and employment/health. Demographic factors (age, education, race, sex) and length between the hurricane and interview date (conducted 2021-2022) were included in analyses. The majority (97%) of our sample was in Puerto Rico at the time of the hurricane. Approximately 41% of participants reported being “somewhat” or “very” frightened following hurricane Maria and over half of participants reported stressors like home repairs, damage to their neighborhood, and concern about bill payment. Regression analyses revealed higher reported fright immediately following hurricane Maria was associated with higher levels of perceived stress at the time of interview (b=.53, p<.001). In addition, all three secondary stressor components were significantly associated with higher perceived stress in separate models (home damage: b=.44, p<.001; family/friends: b=.40, p<.001; employment/health: b=.31, p<.05). Being female was associated with higher perceived stress across each of the regression models. Results suggest long-lasting relationships between hurricane-related stressors and perceived stress several years later, further implicating the role of these stressors on long-term mental and physical functioning.

